# Early versus conventional loading for fully guided immediate implant placement in molar sites: a randomized controlled clinical study

**DOI:** 10.1186/s40729-025-00624-8

**Published:** 2025-05-20

**Authors:** Kirollos H. Botros, Doaa Adel-Khattab, Abdelrahman K. Eldabe, Hala A. Abuel Ela

**Affiliations:** 1 Oral Medicine, Periodontology and Diagnosis, Faculty of Dentistry, Assiut University, Assiut, Egypt; 2 Oral Medicine, Periodontology and Diagnosis Department, Faculty of Dentistry, Ain Shams University, Cairo, Egypt; 3 Oral Medicine, Periodontology and Diagnosis, Faculty of Dentistry, Misr International University, Cairo, Egypt

**Keywords:** Immediate implants, Fully guided, Early loading, Molar area, Resonance frequency analysis, Damping capacity analysis

## Abstract

**Purpose:**

To evaluate early versus conventional loading in immediate implants for molars. This study aims to answer the following PICO (Patient, Intervention, Comparison, and Outcome) question: In patients over 18 years of age, does early loading of immediately placed implants in molar areas result in a similar implant survival rate and marginal bone loss as conventional loading?

**Methods:**

Twenty-seven patients (15 women and 12 men) received a total of 30 implants immediately after molar extraction. The surgical treatment protocol entailed atraumatic tooth extraction without flap elevation. Non-invasive quantitative analyses were used to assess implant stability. After an uneventful healing period, the 30 implants were restored with screw-retained monolithic zirconia prosthesis, half of which after 6 weeks (G1) and the other half after 3 months (G2).

**Results:**

Regarding the survival rate, the Kaplan–Meier and log-rank test showed that there was no statistically significant difference between both groups (*p* = 1). Implant stability quotient at the prosthetic phase of both groups (6 weeks in G1 and 3 months in G2) revealed no statistically significant difference (G1 RFA74.4 (SD 5.54) − DCA 79.07 (SD 5.75))/G2 RFA 73.67 (SD 5.7), − DCA78.93 (SD 4.48).

**Conclusions:**

Early loading of immediately placed implants in molar sites is considered a predictable treatment modality provided that ideal implant position and adequate insertion torque are achieved.

## Background

Restorative therapy performed on implant(s) placed in a fully healed and non‐compromised alveolar process has high clinical success and survival rates [1]. It was proposed that placement of an implant in a fresh extraction socket may stimulate bone tissue formation and osseointegration, and hence counteract the adaptive alterations that occur following tooth extraction. In other words, type 1 implant installation may allow the preservation of bone tissue of the socket and the surrounding jaw [2, 3]. Loading concepts in implant dentistry have been widely discussed in the literature. Initially, healing phases of 3 months in the mandible and 6 months in the maxilla were recommended [4]. To meet the patient’s expectations for earlier prosthetic rehabilitation, shortened healing periods between implant installation and loading have been introduced. A variety of influencing factors, like initial implant stability, implant surface characteristics, bone quantity, bone healing, interim prosthesis design, and occlusal pattern during the healing phase, have been identified for successful osseointegration with modified loading protocols [5]. Developments in the implant design, especially including structured and chemically modified surfaces, have led to the accelerated osseointegration of implants [6–8]. The survival rates of different loading protocols (immediate, early, and conventional) remain a point of debate in the literature. However, the marginal bone loss and peri-implant gingival level are usually not affected by the loading regimen, indicating that different loading protocols behave similarly after successful osseointegration [9–11].

Osseointegration occurs in two stages, the primary and secondary stages [12]. In the primary stage, implant stability is mainly achieved from mechanical engagement with cortical bone. In contrast, in the secondary stage, implant stability is achieved through bone regeneration and remodeling [13]. Adequate primary stability is a prerequisite for acceptable osseointegration. It is, therefore, imperative to quantify implant stability at several time points and predict long-term prognosis based on the obtained implant stability measurements. There are several methods to measure primary stability such as ITVs (insertion torque values). Other techniques involve non-invasive quantitative analysis, such as resonance frequency analysis (RFA) and damping capacity analysis (DCA) [14–16].

It should be pointed out that meta-analysis of published data indicates that while guided computer-assisted implant surgery CAIS is superior to freehand implant surgery in terms of safety and postoperative morbidity, implant success with CAIS is more or less the same [17]. Fully guidance means not only creation of the osteotomy bed through the surgical guide but also fixture insertion that ensures 2 benefits: ideal fixture placement according to the exact planned implant position and adequate insertion torque (primary stability) [18, 19].

Various surgical and prosthodontic protocols used in oral implantology are directly associated with the long‐term outcome of implant prosthesis. A systematic review highlighted the importance of evaluating outcomes in oral implantology by combining the placement and loading protocols variables as a single denominator for survival/success [20].

To the best of our knowledge, no prior clinical trial has compared the outcome of implants following type 1B protocol (immediate placement, early loading) vs type 1C (immediate placement, conventional loading) in molar sites with non-splinted superstructures. The null hypothesis was that there would be no difference in survival rates or marginal bone level changes in immediately placed implants between the two loading procedures, against the alternative hypothesis of a difference.

## Methods

### Patient recruitment

All patients were recruited from the outpatient clinic of Oral Medicine, Periodontology, and Oral Diagnosis Department, Faculty of Dentistry, Ain Shams University, from March 2024 to December 2024. Prior to inclusion into the study, patients were informed about the use of their data for academic purposes and written informed consent was obtained. The study was approved by the Ain Shams Institutional Ethical Committee, Faculty of Dentistry, Ain Shams University (FDASU-RecIM112221). All patients were treated in accordance with regional laws, good clinical practice, and in adherence to the Declaration of Helsinki (1996) [21]. In addition, the study was registered in clinical trial registration site (NCT06281535) on 24/02/2024.

### Sample size calculation

The sample size was calculated based on the primary outcome measure, the implant stability quotient (ISQ). According to a previous study reporting a standard deviation of 3.43 and mean difference of 7, calculations based on an independent-sample-t-test with a power = 90% and a significance level p = 0.05 yielded 12 participants per group. The number was increased to 15 participants (n = 30 in total) to account for any possible dropouts (Attrition Ratio 20%). The adequacy of the sample size used was determined using Ps Power and Sample Size software (version 3.1.2) [22].

### Randomization, allocation concealment, and blinding

Computer-generated randomization lists were created with two groups consisting of an equal number of patients. Only one of the investigators, not involved in the selection and treatment of the patients, was aware of the random sequence and had access to the randomization list stored in a password-protected portable computer. The random codes were enclosed in sequentially numbered, identical, opaque, sealed envelopes. Envelopes were opened sequentially only after implant placement; therefore, treatment allocation was concealed to the investigator in charge of enrolling and treating the patients included in the trial [23].

The inclusion criteria were patients age range from 20–60 years, molar tooth to be extracted due to periodontal diseases, peri-apical diseases, or tooth fracture. Any type of extraction socket (A, B, or C) according to Tarnow and Smith classification provided that insertion torque is above 25 Ncm [24].

The exclusion criteria were extraction sockets that are affected by an acute infection, participants with a history or presence of severe uncontrolled systemic disease, long-term steroidal anti-inflammatory drug therapy, patients exposed to irradiation in the head and neck region with more than 70 Gy within the last 6 months, pregnant or nursing, patients with oral parafunctional (bruxism), heavy smokers (> 10 cigarettes per day) or alcohol/drug abusers, poor oral hygiene, full-mouth plaque surfaces ≥ 10%, patients unwilling to commit to an appropriate post-therapeutic maintenance regimen [25].

### Virtual implant planning and surgical guide fabrication

Cone beam computer tomographies were acquired using Veraview X800 L P (JMorita Mfg. Corp., Kyoto, Japan) with standard settings for all patients (100 kV, 8 mA, 9 s, voxel size: 250 μm, FOV: 110 mm). Concurrently, digital impressions were captured using intraoral scanner Aoral scan3 (shining 3D, China). These impressions accurately represent the surface topography of the dental arches, capturing the details of the gingiva, teeth, and existing prosthetic structures. The high-resolution surface data is essential for designing restorations and planning the prosthetic outcome.

Then, the acquired CBCT and digital impression files were imported into the Exoplan software. The software supports various file formats, such as DICOM for CBCT data and STL for digital impressions. Initial alignment was achieved by manually selecting corresponding anatomical landmarks. Common reference points include cusp tips, incisal edges, and occlusal grooves. The software’s intelligent algorithms assist in aligning these points to create an initial match between the datasets. Once the initial alignment is established, Exoplan’s advanced registration algorithms perform a fine-tuning process. The prosthetic-driven planning approach in Exoplan allows for the consideration of both surgical and prosthetic factors, ensuring that the implant positions align with the desired restorative outcomes.

**Fig. 1:**
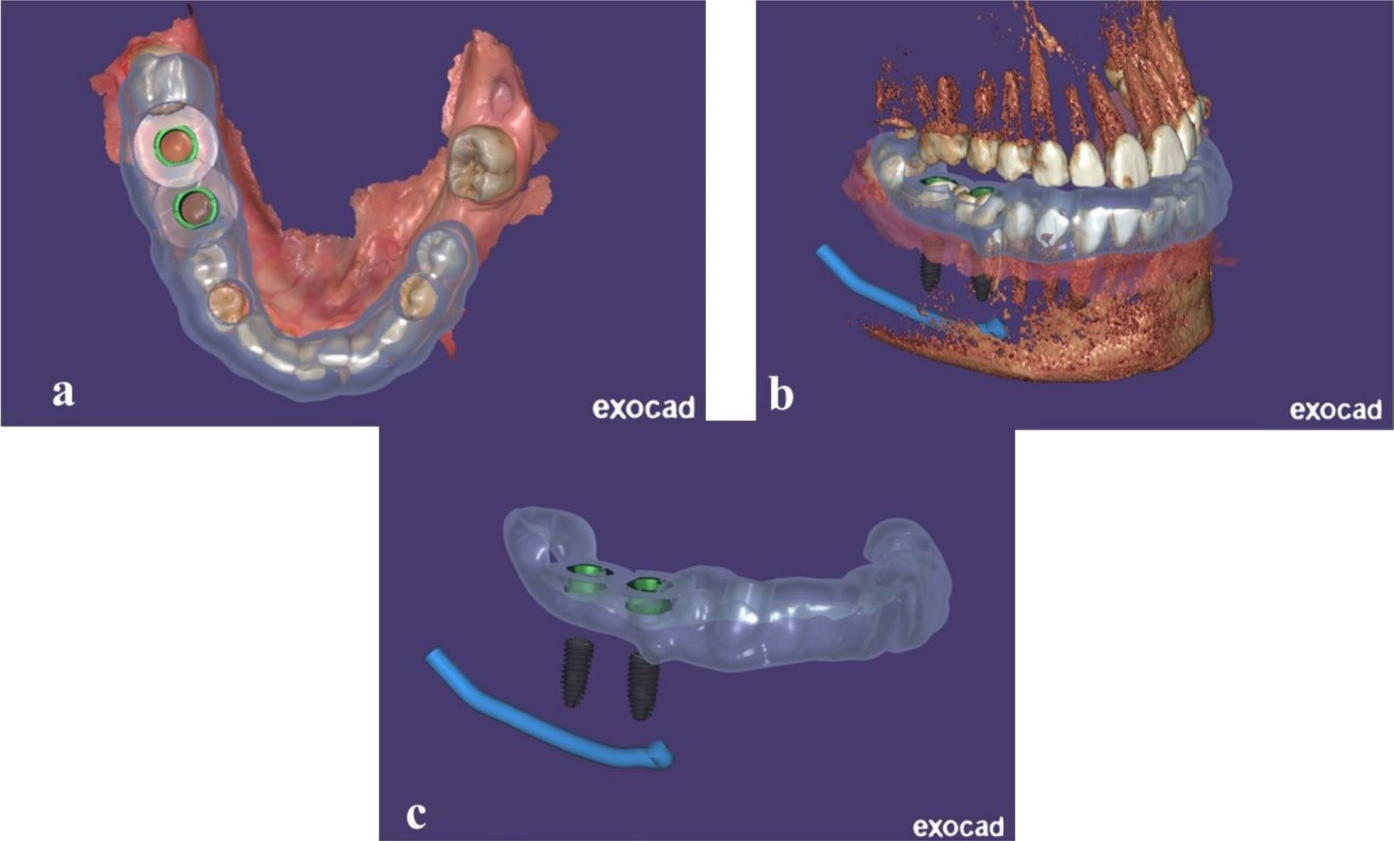
**a**–**c** Designing a fully guided surgical guide to translate the digital plan into precise clinical execution

The finalized treatment plan, incorporating the aligned datasets, was used to design fully guided surgical guides (Fig. 1). The fully guided surgical guide dictates the exact trajectory of the surgical drills and implants, translating the digital plan into precise clinical execution and ensuring that the implant placement adheres to the meticulously planned positions. These guides were fabricated using biocompatible materials and DLP 3D printer Accuafab D1s (shining 3D, China).

### Intervention (surgical procedure)

Before the surgical procedure, Phase 1 Therapy was done to improve the oral environment for better wound healing. All patients would receive prophylactic antibiotic therapy: 1 g of (amoxicillin + clavulanic acid ·1000 mg b.d.s) (Amoxil MUP Egypt) every 12 h from the day before the surgery to the sixth post-surgical day. Patients rinsed with 0.2% chlorhexidine mouthwash (Antiseptol Kahira Pharm Egypt) for 1 min prior to any intervention. Local anesthesia was achieved using Articaine HCL 4% (Septodont LTD, Septanest 1:100,000) [26].

**Fig. 2:**
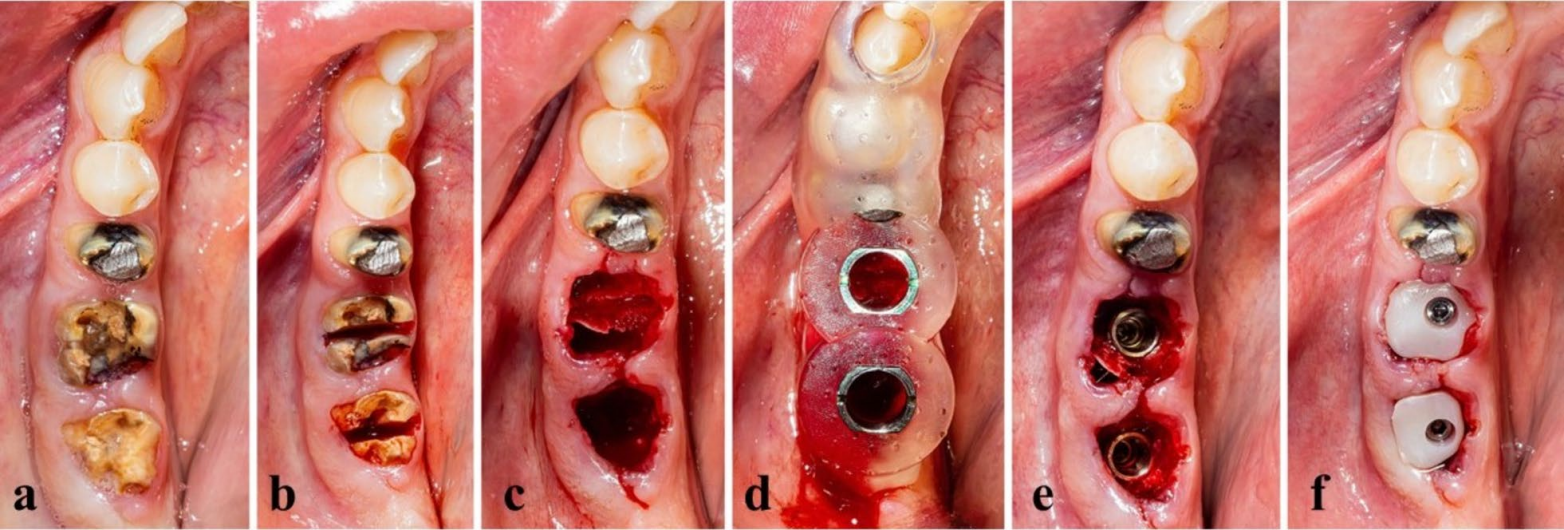
**a**–**f** The steps of the surgical procedure, including atraumatic extraction, fully guided fixture placement, followed by placement of a prefabricated customized healing abutment

The surgical treatment protocol entailed atraumatic tooth extraction without flap elevation, thereby maintaining the periosteal blood supply. The extraction socket was debrided of any granulation tissue, and rinsing with saline solution was done. Fully guided implant placement would be performed. In other words, not only was the osteotomy made through the guide, but also fixture insertion, thus ensuring two main benefits: one is ideal fixture placement without any slippage, and the other is to guarantee a high insertion torque (Fig. 2).

Implants were inserted in the osteotomy site with the motor set at a torque of 30 Ncm and, once the motor stopped, implants were placed manually with a ratchet (Neobiotech) until they were at the same level as the inter-septal bone. The wrench used was able to perform torque measurements within a range of 15–80 Ncm, with 5% precision. In case an implant was inserted with a torque inferior to 30 Ncm, a larger diameter implant would be used in order to obtain the required insertion torque, or to be excluded from the research [23, 25–27].

After having completed the implant placement procedure, screw-retained prefabricated custom healing abutments were utilized. They were milled from polymethyl methacrylate (PMMA) blanks using a 5-axis milling machine with an accuracy of repetition equal to 1 micron.

These Customized healing abutments were designed using Dental CAD software (Exocad, Darmstadt, Germany) and had the same natural emergence profile of the tooth to be extracted. The workflow involved the combination of intraoral and CBCT scans, from which three-dimensional (3D) reconstructions of soft tissue and alveolar bone, as well as of the tooth to be extracted were obtained and exported as STL files. The files were imported to the computer-aided design (CAD) software, in which a virtual wax-up and custom abutment design was performed considering the natural emergence profile of the patient’s tooth prior to extraction [28]. Sequentially numbered envelopes corresponding to the patient were opened to know when to load the implant, either early (6 weeks) or conventionally (3 months).

Postoperative care recommendations included 1 g of Amoxicillin and clavulanic acid every 12 h (this antibiotic regimen started 1 day before the surgery). Analgesics, mainly ibuprofen, up to 600 mg every 6–8 h as needed, or Paracetamol 1 g for patients allergic to NSAIDs. Patients were advised to rinse the mouth with 0.12% chlorhexidine gluconate twice daily for 2 weeks. A soft diet was recommended for 1 week. No prosthesis that compressed the implant would be used during the entire implant healing period [29].

### Prosthetic procedure

**Fig. 3:**
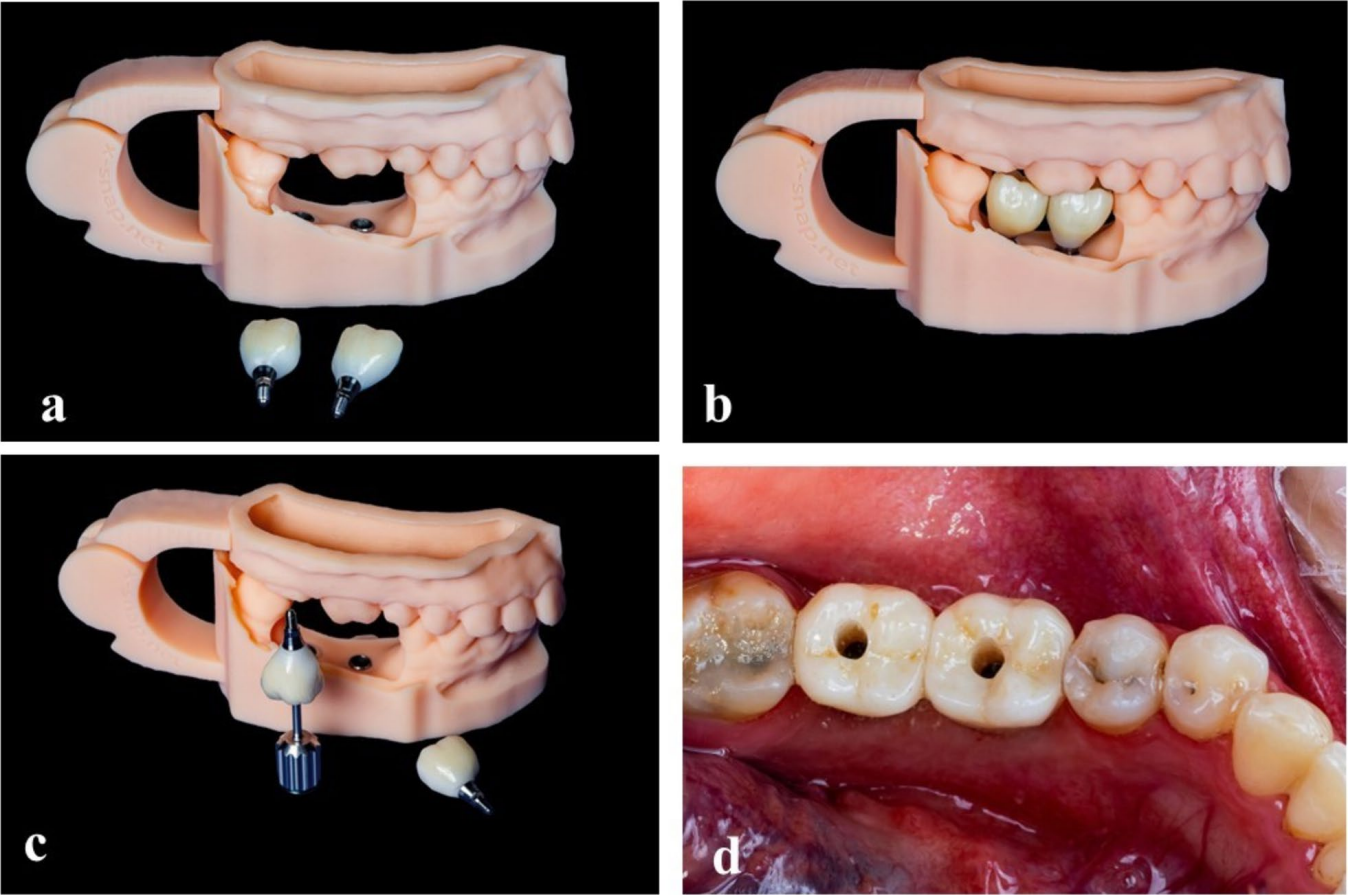
**a**–**c** Monolithic zirconia glued on a titanium base utilizing a 3D-printed model. **d** Non-splinted screw-retained crowns were delivered to the patient

At the end of the healing period, which was normally 6 weeks for the first group and 3 months for the second group, all patients received their final rehabilitation, i.e. occlusal loading restorations. Digital impressions were taken by a blinded operator. Deviations from these periods would only occur when patients were not able to make an appointment within the scheduled week, but this was limited to 5 days deviation; otherwise, the patient would be excluded from the research. Prosthodontic treatment was performed according to the manual provided by the manufacturer. All patients would receive monolithic zirconia screw-retained fixed prostheses (Fig. 3).

## Data processing and assessment

### Implant stability measurements

**Fig. 4:**
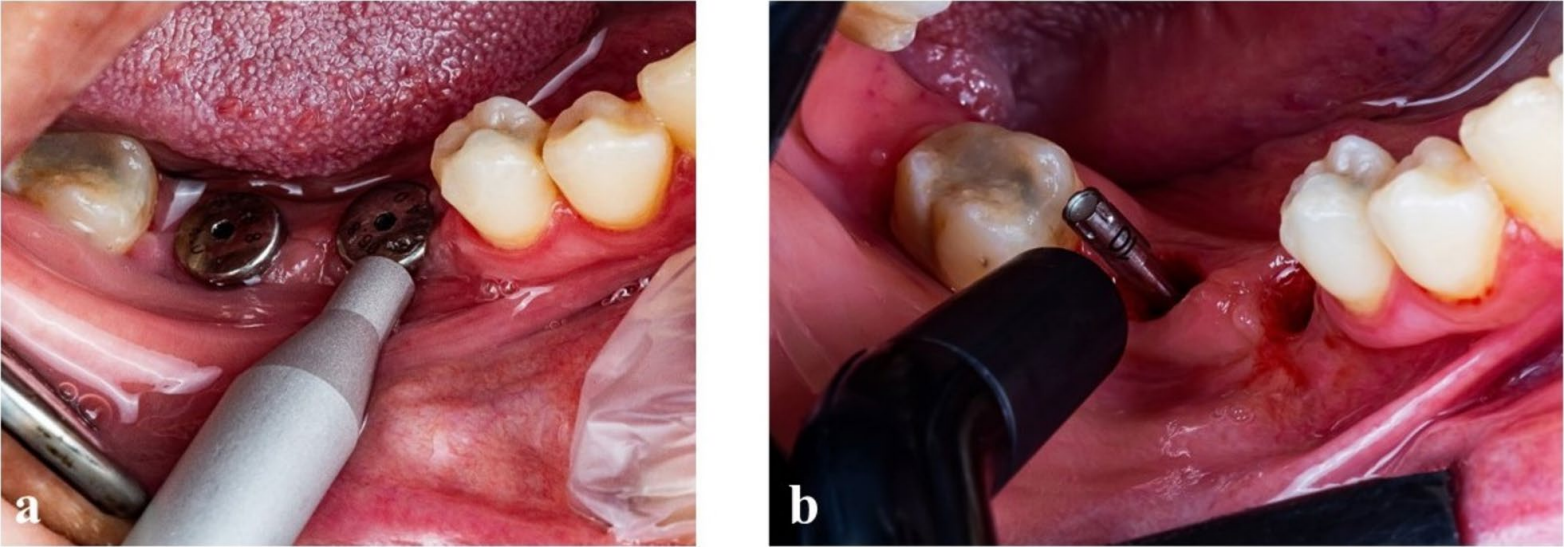
**a**, **b** Anycheck (damping capacity analysis) and Penguin (resonance frequency analysis) were used to measure the implant stability quotient

In both groups, non-invasive quantitative analysis, such as resonance frequency analysis (RFA) and damping capacity analysis (DCA) were used to assess implant stability which was checked instantly after implant placement (T0) for both groups, 6 weeks after surgery (T1) for the first group and 3 months after surgical procedure for the second group (T2). To avoid unscrewing and re-screwing the implant superstructures (healing abutments of group 2 at (T1) and permanent crowns of group 1 at (T2), the implant stability was measured only at the time of implant scanning by scan bodies on the prosthetic phase. Two devices were used: Anycheck (Neobiotech, Seoul, Korea), which is a damping capacity method device that measures the time of contact between the impacting rod and the healing abutment. It strikes the healing abutment six times over three seconds and converts the time into the implant stability test (IST) values. This device strikes the healing abutment with less force compared to the Periotest M and has a function to stop automatically when the stability is low, to protect the implant [30]. At 6 weeks after surgery (T1) for the first group and 3 months after surgical procedure for the second group (T2), the custom healing abutment is unscrewed, and a stock healing abutment with a standard height of 4 mm is tightened to guarantee a correct and reliable iST value. Another device, Penguin RFA, uses a sensor (autoclavable multi peg) coupled with an implant fixture and measures resonance frequency values that are converted into implant stability scale values called the implant stability quotient (ISQ) (Fig. 4).

### Radiographic assessment

MBL was defined as loss, in an apical direction, of alveolar bone marginally adjacent to the dental implant, in relation to the marginal bone level initially detected after the implant was surgically placed.

**Fig. 5:**
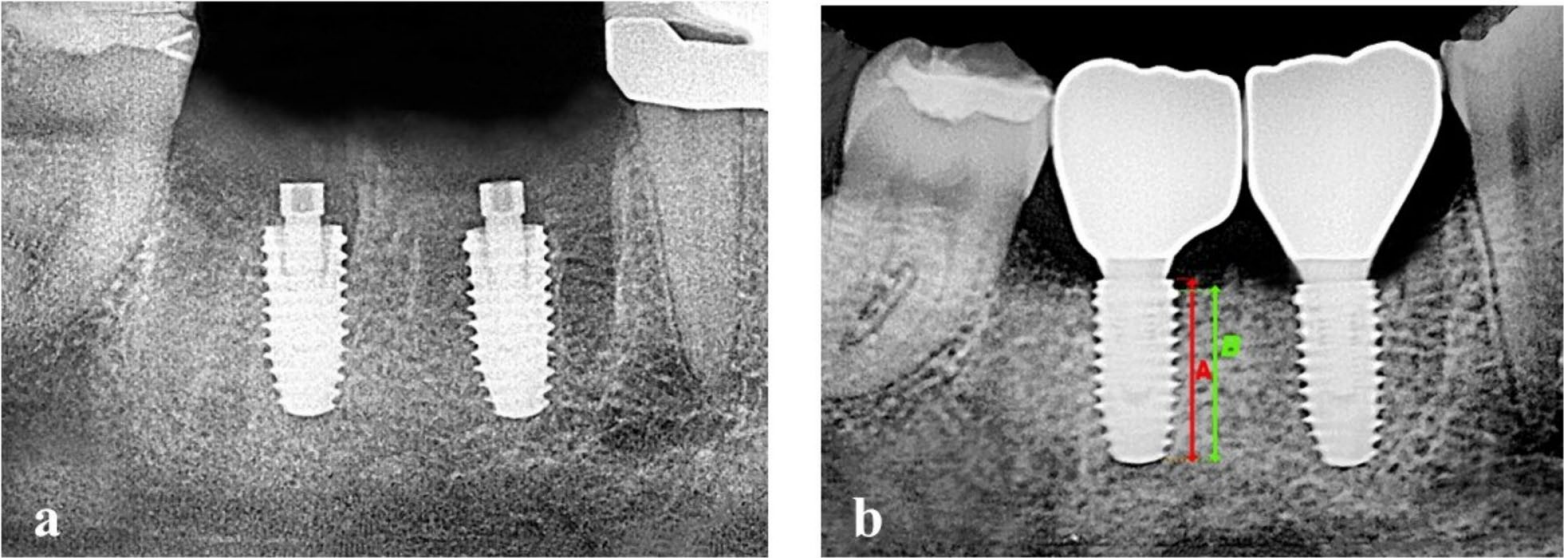
**a**, **b** Periapical X-ray using long cone parallel technique and a phosphor imaging plate instantly after the surgical procedure (T0) and at 12 months follow-up to measure marginal bone loss (MBL)

Patients in both groups will receive periapical x-ray instantly after the surgical procedure (T0) and 12 months later. The process of periapical radiograph acquisition at the clinic is standardized, using the long cone paralleling technique. The reference points for the measurements included the implant platform (the horizontal interface between the implant and the abutment), the implant apex, and the first bone-implant contact (FBIC). The length from the implant platform to the FBIC was defined as the marginal bone level. The marginal bone level was measured in mm using the ratio of the real implant length and the length from the implant platform to the apex on the images. Measurements were taken at both mesial and distal sides of each implant, and then the mean value of these two measurements was considered. The sets of radiographs for every patient were coded, and the doctor who performed the radiological measurements was blinded (Fig. 5).

### Patient satisfaction

At delivery of definitive crowns and 12 months after loading, the local blind outcome assessors provided a mirror to the patients showing the implant-supported crown on which patients were asked to express their opinions. Specifically, the patients were asked, ‘Are you satisfied with the function of your implant-supported tooth?’ Possible answers were ‘yes absolutely’, ‘yes partly’, ‘not sure’,’ not really’, and ‘absolutely not’. Then they were asked, ‘Are you satisfied with the aesthetic outcome of the gums surrounding this implant?’. Possible answers were ‘yes absolutely’, ‘yes partly’, ‘not sure’, ‘not really’, and ‘absolutely not’. Finally, patients were asked whether they would undergo the same therapy again. Possible answers were ‘yes’ or ‘no’. The questions were always posed with the same wording.

The Primary outcome was the implant stability (the degree of osseointegration) measured by two methods: Damping capacity assessment by Anycheck (Neobiotech, Seoul, Korea) and Resonance frequency analysis by Penguin from Klockner.

The Secondary outcomes were the peri-implant marginal bone level changes and Patient Satisfaction. Implant stability and marginal bone level changes were assessed by a blinded outcome assessor. Any complications and adverse events would be recorded [19].

### Statistical analysis

The statistical analysis was performed by an independent statistician who was not involved in the patient selection and enrollment process or the surgical procedures. The chi-square test was used to compare dichotomous variables (failures and complications). An unpaired Student’s t-test was used as a test for differences in RFA, DCA, and MBL values between the trial arms. All statistical analyses were carried out by using a statistical software program (SAS version 9.4; SAS Institute).

## Results

**Fig. 6:**
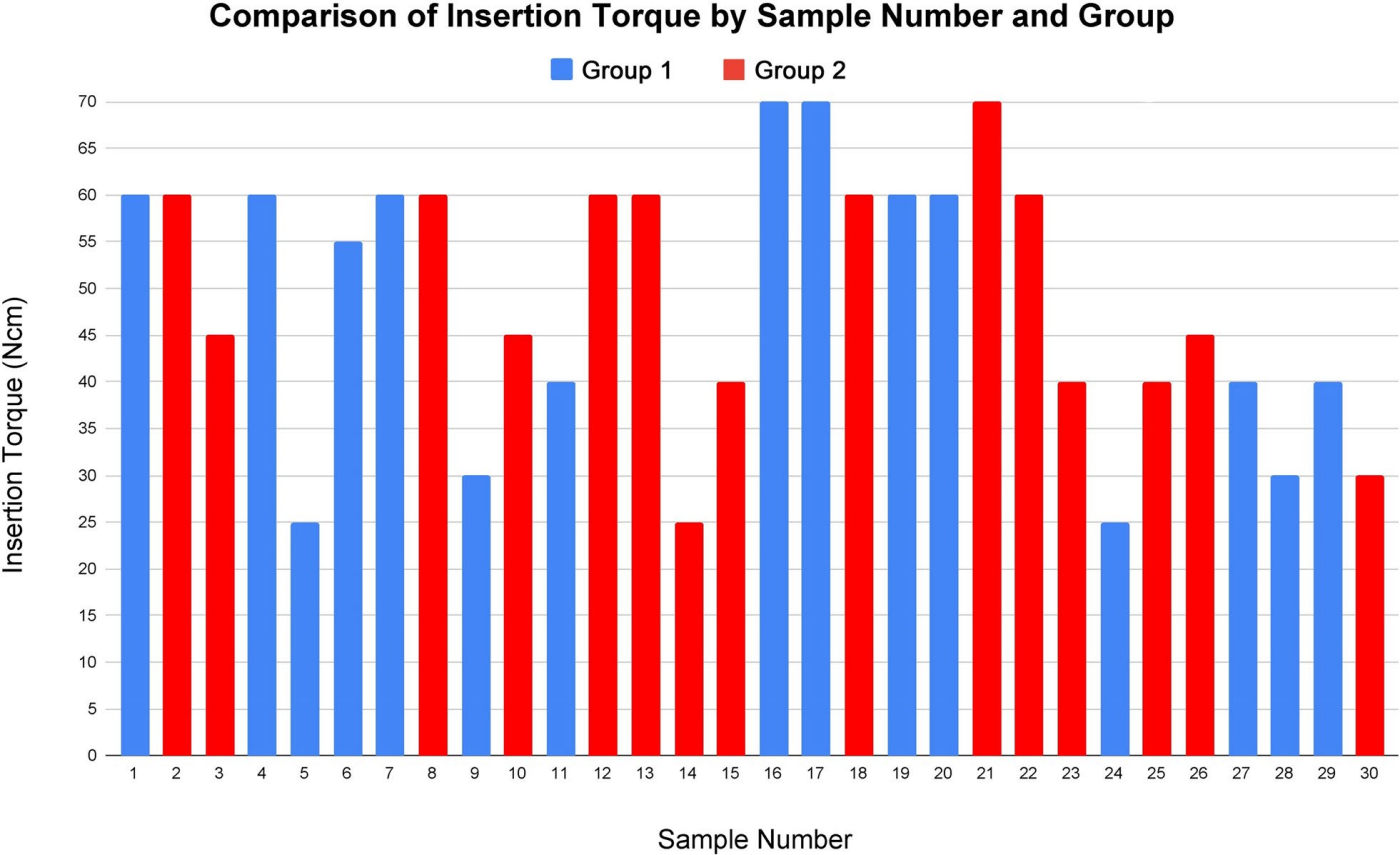
A bar chart shows the insertion torque of each implant in both groups

**Fig. 7:**
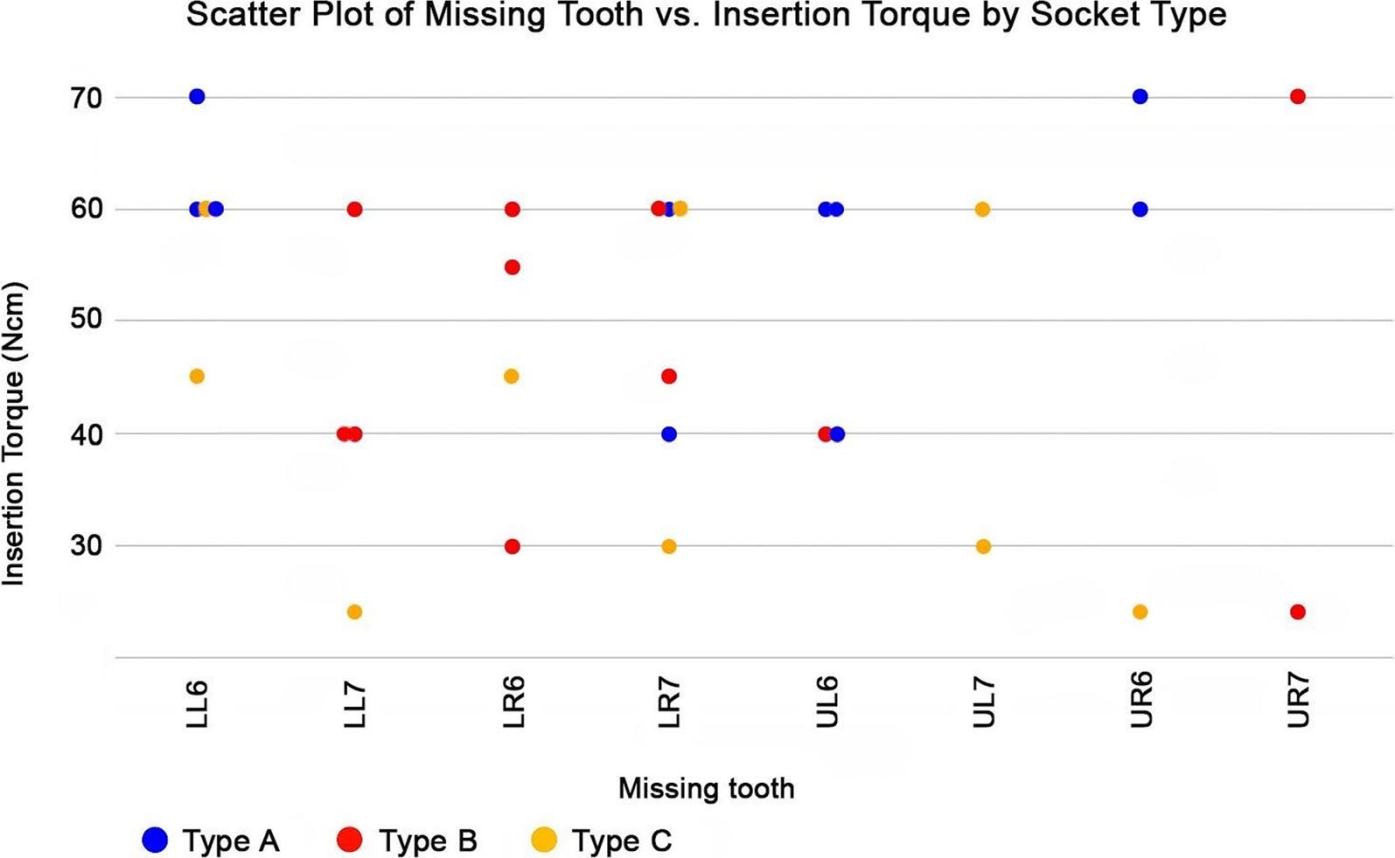
A scatterplot shows missing teeth, insertion torque, and socket type

**Fig. 8:**
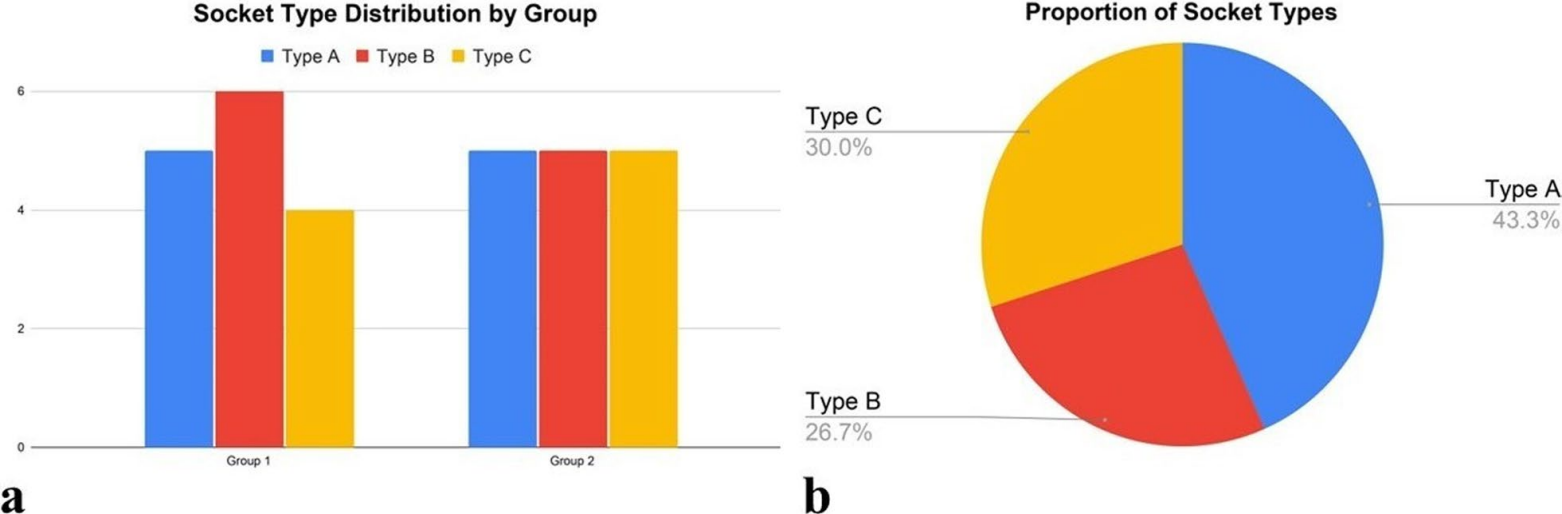
**a** A bar chart illustrates socket type distribution by group. **b** A pie chart shows the proportion of socket types

Twenty-seven patients (15 women and 12 men) received a total of 30 implants immediately after molar extraction. The implanted sites were 7 maxillary first molars, 4 maxillary second molars, 9 mandibular first molars, and 10 mandibular second molars (Figs. 6, 7 and 8). After an uneventful healing period, the 30 implants were restored with screw retained monolithic zirconia prosthesis, half of which after 6 weeks (G1) and the other half after 3 months (G2). Normality was checked with QQ plots, histograms, and the Shapiro–Wilk test. The Shapiro–Wilk test suggests that the data does not significantly deviate from normality.

**Fig. 9:**
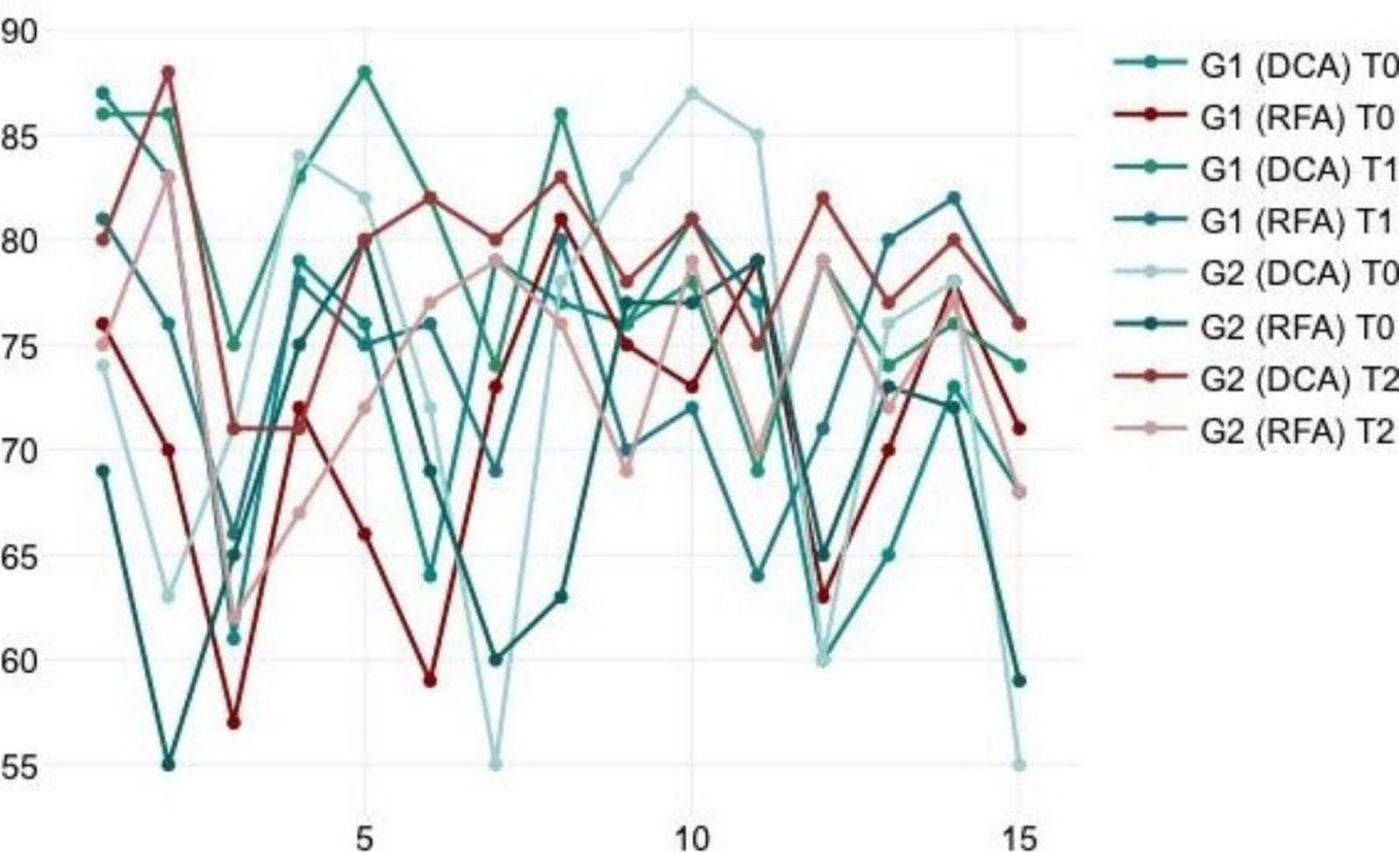
A line graph displays the analysis of resonance frequency and damping capacity at various time intervals for both groups

A log-rank test was calculated to see if there was a difference between the two groups, Early loading (G1) and Conventional loading (G2), in terms of survival rate. For the present data, the Kaplan–Meier and log-rank test showed that there is no statistically significant difference between both groups, p = 1. Therefore, the null hypothesis is not rejected (Fig. 9).

In the test group (G1), the average RFA values increased from 70.87 (SD 7.04) at the time of implant placement (T0) to 74.4 (SD5.54) after 6 weeks (T1). Also, the average DCA values increased from 73.73 (SD 8.27) to 79.07 (SD 5.75). Similarly, in the control group, the average RFA and DCA values were 69.2 (SD 7.81) and 73.53 (SD 10.76), respectively, at the time of implant placement (T0). After 3 months (T2), both increased to 73.67 (SD 5.7) and 78.93 (SD 4.48) (Table 1).

**Table 1:** Shows the values of RFA and DCA for each implant in both groups

Number	Group	Implant stability quotient					
		T0		T1 (G1)		T2 (G2)	
		DCA	RFA	DCA	RFA	DCA	RFA
1	G1	87	76	86	81		
2	G2	74	69			80	75
3	G2	63	55			88	83
4	G1	83	70	86	76		
5	G1	61	57	75	66		
6	G1	79	72	83	78		
7	G1	76	66	88	75		
8	G2	71	65			71	62
9	G1	64	59	82	76		
10	G2	84	75			71	67
11	G1	79	73	74	69		
12	G2	82	80			80	72
13	G2	72	69			82	77
14	G2	55	60			80	79
15	G2	78	63			83	76
16	G1	77	81	86	80		
17	G1	76	75	76	70		
18	G2	83	77			78	69
19	G1	81	73	78	72		
20	G1	77	79	69	64		
21	G2	87	77			81	79
22	G2	85	79			75	70
23	G2	60	65			82	79
24	G1	60	63	79	71		
25	G2	76	73			77	72
26	G2	78	72			80	77
27	G1	65	70	74	80		
28	G1	73	78	76	82		
29	G1	68	71	74	76		
30	G2	55	59			76	68

When comparing the mean of both RFA and DCA measurements between the control (G2) and the test group (G1), an unpaired Student’s t-test revealed no statistically significant differences (*p* = 0.724) (*p* = 0.944), respectively.

**Fig. 10:**
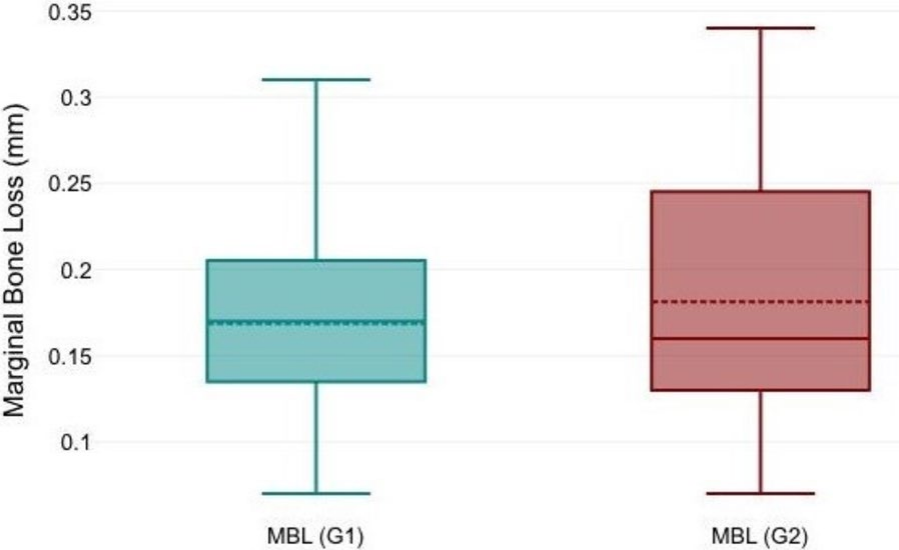
A box plot describing the marginal bone level changes in both groups

The radiographic analysis showed stable bone conditions with a maximum bone loss of 0.34 mm. The early loaded implants did not show different mean values from conventionally loaded implants after 12 months (test 0.17 mm, SD 0.07; control 0.18 mm, SD 0.08). The difference was statistically insignificant (*p* = 0.639). Applying the Albrektsson radiographic success criteria, the overall implant success rate was 100% (Table 2) (Fig. 10).

**Table 2:** Shows the mean, standard deviation, minimum and maximum of RFA, DCA and MBL

	G1					G2				
	DCA (T0)	RFA (T0)	DCA (T1)	RFA (T1)	MBL (mm)	DCA (T0)	RFA (T0)	DCA (T2)	RFA (T2)	MBL (mm)
Mean	73.73	70.87	79.07	74.4	0.17	73.53	69.2	78.93	73.67	0.18
Std. deviation	8.27	7.04	5.75	5.54	0.07	10.76	7.81	4.48	5.7	0.08
Minimum	60	57	69	64	0.07	55	55	71	62	0.07
Maximum	87	81	88	82	0.31	87	80	88	83	0.34

Patient satisfaction was assessed at the time of final prosthesis delivery (6 weeks for group 1 and 3 months for group 2) and at 12 months after loading. Regarding the function of the prostheses at the time of delivery, 13 patients of group 2 stated they were absolutely satisfied versus 11 patients of group 1; one patient from each group declared to be partially satisfied, and one patient from group 1 declared to be uncertain about the function of his crown. At 12 months after loading, 11 patients of the group 2 declared to be absolutely satisfied and three were partially satisfied versus all 13 patients who were absolutely satisfied in group 1. Regarding aesthetics, at the time of loading, all patients of both groups declared to be completely satisfied. At the 12-month follow-up, 12 patients of group 2 were absolutely satisfied and two were partially satisfied versus 12 patients who were absolutely satisfied and one who was partially satisfied in group 1. There was no statistically significant difference regarding function and aesthetic satisfaction between both groups (function satisfaction at time of delivery: *p* = 0.375, function satisfaction at 12 months follow up: *p* = 0.336; aesthetics satisfaction at time of delivery: *p* = 0.337, aesthetics satisfaction at 12 months follow up: *p* = 0.583). All patients declared that they would undergo the same procedure again, both at the time of prosthesis delivery and 12 months after loading.

## Discussion

The primary outcome of this randomized, two-armed clinical trial was to compare implant stability between early (6 weeks) and conventionally loaded (3 months) implants placed immediately in fresh extraction molar sockets. As the survival rate was 100% for both groups and there was no statistical difference regarding implant stability (RFA, DCA), the first null hypothesis was accepted. The second null hypothesis was also accepted because there was no statistical difference regarding marginal bone loss or patient satisfaction between the groups.

The insertion torque (IT) of the implant into the bone significantly affects the outcome of implant therapy; thus, the capability of some equipment to quantify and assess implant stability in an accurate manner has dramatically enhanced the efficacy and quality of implant procedures. The Penguin gadget measures the implant stability quotient (ISQ) by resonance frequency analysis (RFA), while the Anycheck device uses the percussion approach. In a recent study, Okuhama et al. compared the measures of the RFA device (Osstell) with damping capacity (Anycheck) in 7 patients with 15 implants. It was the first clinical study to conduct this comparison on a weekly basis for 6 weeks. There was a positive correlation between all measurement points (r > 0.5) except week 1, with Anycheck usually providing slightly elevated values. The authors indicate comparable performance of both devices and highlight the superiority of Anycheck by eliminating the need to remove the healing abutment for each measurement, unlike RFA systems [31]. A lot of authors explain the risk of frequent removal of healing abutments and its role in crestal bone resorption [32, 33].

The time factor is very important for the patient when considering implant procedures. Immediate implant placement in the esthetic zone is a very common operation and is usually executed with a high survival rate, reaching 98.4% at 2 years follow-up [34]. Regarding the molar area, immediate placement usually seeks a more experienced operator due to the morphology of the socket after extraction and the closer to vital structures (Inferior alveolar canal and maxillary sinus). Tarnow and Smith classify implant placement into molar sockets and indicate high predictability and success rate when initial implant stability is achieved within the septal bone, either completely (type A socket), partially (type B socket), or by engaging the peripheral walls of the socket (type C socket). Should primary stability be unattainable, or in the absence of the buccal plate of bone for implant stabilization, a delayed procedure must be employed [24].

A systematic review conducted by Esposito et al. [10], including 11 randomized trials and comparing the 3 protocols of implant loading (immediate vs. early vs. conventional loading), revealed no statistically significant differences between the 3 regimes for any of the parameters tested. Outcome measures were prosthesis failures, implant failures, and marginal bone levels. The authors highlighted the role of the operator's experience and high implant primary stability as prerequisites for a successful immediate/early loading procedure. Bone quality and quantity, implant macro design (thread depth and pitch), and surgical technique are among the factors affecting primary stability. A recent systematic review and meta-analysis demonstrated that implants placed in fresh extraction sockets at molar sites achieved survival rates of 96.6% during a minimum follow-up period of 1 year. The authors emphasize the significance of atraumatic extraction, a flapless technique, and primary stability to achieve a high success rate, minimize postoperative peri-implant tissue loss, reduce operative time, expedite post-surgical healing, diminish postoperative complications, and enhance patient comfort [35].

In the present study, the survival rate was 100% in both groups (either early loaded or conventionally loaded) until 1-year follow-up. The obtained results are in line with a recent study with long-term follow-up (3 years minimum) showing 96% survival and success rate with only one implant failed from 25 implants placed immediately in the molar site. All the implants were loaded conventionally (after 6 months of the surgical operation). The authors justify the cause of failure to socket infection and improper occlusion in this site [36]. Contrary to these results, one study showed a high failure rate (73.3% survival rate) for immediate implants in molar sites. The authors highlight the role of implant macro design ( large thread depth with sharp thread edges and a small thread pitch) and implant diameter to have a large contact area with the socket bone and to attain adequate primary stability in various socket morphologies [37].

In a systematic review by German Gallucci, 12 different intervention protocols, depending on the time of implant placement (immediate, early, and delayed) and time of loading (immediate, early, and conventional), were compared. The study revealed no scientific validation for some interventions, including type 1B procedure (Early loading of immediately placed implants) [20]. In the present study, the early-loaded group showed the same outcomes as conventional loading with no statistical difference regarding survival rate (100% survival rate of both groups), implant stability at the prosthetic phase (G1 RFA 74.4 ± 5.54, DCA 79.07 ± 5.75 vs G2 RFA 73.67 ± 5.7 and 78.93 ± 4.48) and marginal bone loss (G1 MBL 0.17 mm vs G2 MBL 0.18 mm). Esposito et al. [38] in a multi-center study compared immediate, early (6 weeks), and delayed loading (3 months) of single, partial, and full arch implant-supported prostheses, whether the implants were placed immediately in fresh extraction sockets or delayed in healed ridges. The results correspond well with our research, indicating preimplant marginal bone loss of 0.19 ± 0.44 mm for immediately loaded implants, 0.18 ± 0.66 mm for early loaded implants, and 0.25 ± 0.28 mm for conventionally loaded implants. No statistically significant differences were seen in complications (P = 1.000) and bone loss (P = 0.806) across the three loading techniques. Also, no instances of implant failure and few complications were documented; hence, all three procedures appear to be highly effective. In the previous study, the authors rationalized the good results obtained (100% success rate) to the high insertion torque. They stipulate plus 40 Ncm insertion torque for immediate and early loading protocols; otherwise, the loading will be delayed ( after 4 months).In instances of medium and soft bone quality, implant sites were inadequately prepared with drills that were one or two sizes smaller than the final implant diameter [38–40].

To our knowledge, this is the first clinical study that compares early loading to conventional loading in immediately placed implants in molar sites with non-splinted prostheses. The limitations of the study are the short follow-up period, the lack of investigation of soft tissue around the implant sites, and no homogeneity between the sockets included in the study.

## Conclusion

Considering the limitations of this study, early loading of fully guided immediately placed implants in molar sites may be considered a predictable treatment modality provided that ideal implant position and adequate insertion torque are achieved.

## Data Availability

Data is available upon reasonable request.

## Abbreviations

ITVs: Insertion torque values
RFA: Resonance frequency analysis
DCA: Damping capacity analysis
FBIC: First bone-implant contact
CAIS: Computer-assisted implant surgery
ISQ: Implant stability quotient
DLP: Digital light processing
PMMA: Polymethyl methacrylate
STL: Standard tessellation language
